# Smouldering wildfires in peatlands, forests and the arctic: Challenges and perspectives

**DOI:** 10.1016/j.coesh.2021.100296

**Published:** 2021-12

**Authors:** Guillermo Rein, Xinyan Huang

**Affiliations:** 1 Department of Mechanical Engineering, Imperial College London, UK; 2 Research Centre for Fire Safety Engineering, The Hong Kong Polytechnic University, Hong Kong, China

**Keywords:** Peat, Forest, Firebrand, Pollution, Safety, Emissions, Climate change

## Abstract

Wildfires can be divided into two types, flaming or smouldering, depending on the dominant combustion processes. Both types are present in most wildfires, and despite being fundamentally different in chemical and physical terms, one transitions to the other. Traditionally, science has focused on flames, while smouldering is often misinterpreted. But smouldering wildfires are emerging as a global concern because they cause extensive air pollution, emit very large amounts of carbon, are difficult to detect and suppress, and could accelerate climate change. Central to the topic are smouldering peat fires that lead to the largest fires on Earth. Smouldering also dominates the residual burning after flames have died out and firebrand ignition. Finally, smouldering is an important part of Arctic wildfires, which are increasing in frequency. Here, we present a scientific overview of smouldering wildfires, the associated environmental and health issues, including climate change, and the challenges in prevention and mitigation.

## Smouldering wildfires

Wildfire is a natural phenomenon that shapes ecosystems globally, interacts with the climate and threatens human communities [1]. As a combustion process, fire can be broadly divided into two types, flaming or smouldering [2]. Both are present in most wildfires, and despite being fundamentally different in chemical and physical terms, one can transition to the other [3]. Tradition in fire science has focused on flames, while smouldering remains poorly studied and often misinterpreted. This paper focuses on smouldering wildfires.

Smouldering combustion is the slow, low temperature and, flameless burning of a solid fuel involving heterogeneous chemical kinetics [4]. The fire spread through the peat layer is sustained by the heat released when oxygen directly reacts with the surface of soil particles. While flaming combustion is the fast and high temperature burning of a gaseous fuel involving homogenous chemical kinetics [5]. The typical temperature of smouldering is relatively low, around 500 °C. Smouldering wildfire spreads in a creeping fashion, typically around 1 cm/h, which is two orders of magnitude slower than the spread rate of flaming fires.

**Figure 1:**
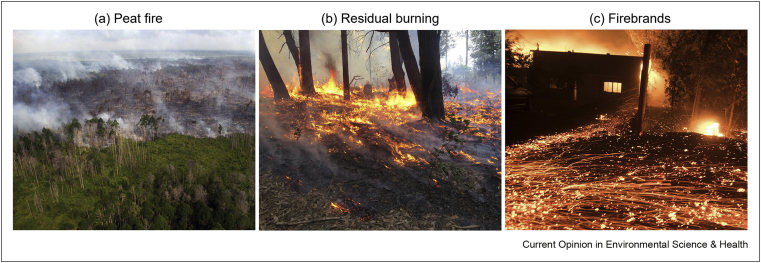
Primary forms of smouldering wildfires, (**a**) peat fire of deep layers, (**b**) residual burning after the flames and (**c**) firebrand attack on houses and vegetation. Credits: Reuters 2017, YubaNet 2018, and UPI Barcroft Media 2020.

Smouldering is the most persistent type of combustion because it is easier to ignite but more difficult to suppress than flaming combustion [2,3]. In fact, the longest burning fire on Earth is a smouldering coal seam known as the *Burning Mountain* in New South Wales, Australia, which ignited more than 6000 years ago [6]. In wildfires, it primary appears in three forms (see Figure 1), (a) peat fire, where the soil burns deep layers for long periods of time and contributes to haze, (b) residual burning, where thicker vegetation burns after flames have passed, and white smoke is emitted and (c) firebrands, when the burning debris from wildfires become airborne and attack remote locations.

Driven by anthropogenic effects and climate change, peat fires (Figure 1a) occurs at an increasing frequency in all regions where histosols are found, from tropical wetlands to the Arctic [7, 8∗, 9, 10∗∗, 11∗∗]. Peat fires can reach very large sizes and burn vast amounts of fuel many times more than flaming fires [2]. For example, the smouldering wildfires in the peatlands of Indonesia can last for weeks and produce extensive air pollution episodes, called haze, that annually affect millions of people [12]. Overall, smouldering wildfires are emerging as a global scientific and societal concern because of their extensive air pollution, enormous carbon emissions, difficulty in suppression, and contribution to climate change.

After the flames of a wildfire pass by, often, forest fuels continue burning in the form of smouldering (Figure 1b). The disappearance of flames can create a false negative for wildfire extinction, while smouldering continues to spread, consuming fuel and releasing toxic gases. Firebrands (Figure 1c) are burning embers generated from wildfires, typically twigs and barks, that become airborne in the plume and are carried away by the wind [13,14]. The glowing of the firebrand indicates smouldering combustion and is especially visible at night. Small firebrands can fly for hundreds of metres, transitioning to flaming ahead of the main wildfire, breaching firebreaks, and attacking remote locations.

In this article, we provide a scientific overview of smouldering wildfires. Our emphasis is on large peat wildfires and the associated environmental and health issues, including climate change and the challenges of mitigation.

## Large peat fires

Smouldering combustion is the dominant burning process of wildfires in peatlands [2,3]. While flames consume the surface vegetation, smouldering consumes the histosol. Smouldering megafire were brought to the forefront of scientific debates with the studies of the 1997 extreme haze event in Southeast Asia from burning peatlands [8]. The smoky haze covered large parts of Southeast Asia, even reaching parts of Australia and China. It induced a surge of respiratory emergencies in the population and disruption of shipping and aviation routes for weeks. This single event led to the approval of the ASEAN Agreement on Transboundary Haze Pollution in 2002.

Smouldering peat wildfires frequently occur worldwide in tropical, temperate, and boreal regions. Droughts, drainages, and changes in land use and population density are the main causes [15]. Europe's most affected ecosystems are the temperate and boreal forests in Russia, British Isles, and Scandinavia [16, 17, 18]. Other affected regions in the rest of the world are North America (e.g. Canada, Alaska and Florida), southern Australia and Central Africa [7]. A large portion of peat fires in Southeast Asia is ignited by the practice of slash-and-burn that quickly clears vegetation for plantation [19,20]. Other possible ignition causes of peat fire can be natural, such as lightning and self-heating ignition [21].

Peatlands often contain high water tables, so peat is usually too wet to support burning. However, the water table of peatlands can descend, and the moisture content of organic soils is the single most important property governing the ignition and spread of peat wildfires. For example, the critical moisture content for igniting typical boreal peat samples has been measured at about 125% in the dry base where the soil mineral content is less than 10% [22, 23, 24, 25]. Smouldering fires propagate horizontally and vertically through organic layers of the ground and can reach deep into the soil where large cracks or natural piping systems exist.

In general terms, flaming fires produced substantial flame heights but minimal heating to the soil [26]. In contrast, smouldering fires produce many times longer heating duration (more than 1 h) and reach lethal temperatures of most species [22,27]. The longer duration and the greater heat transferred to the forest floor by smouldering has been identified as an important factor in wildfire mortality [28]. Moreover, these underground fires can release ancient carbon (>10,000 years old) stored in the soil.

**Figure 2:**
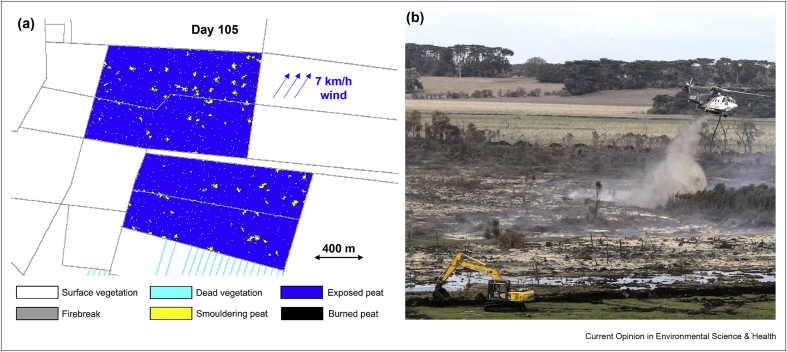
(**a**) Simulation of smouldering peat fire of 573 ha in Indonesia triggered by the prescribed fire [29] and (**b**) fighting and control the peat wildfire (Gunstone, CC-BY).

Recent developments in physical modelling are improving the understanding of the ignition and extinction limits of smouldering peat [30, 31∗, 32]. Cellular Automata modelling has been applied recently to simulate peat fires at the field scale [29,33] (Figure 2a). In the near future, a peat fire simulator (similar to the flaming simulator FARSITE [34]) should be developed for smouldering, combining fuel, water table and wind information based on GIS. Unlike simulating flaming wildfires, the peat fire simulator should include the additional dimension of depth to visualise the 3D spread processes in the ground.

The detection of peat fires by satellite is challenging because the fire might be partially underground, and the radiant signature is different and weaker than from flaming. Deep peat wildfires can even take place several metres deep, so they are different, to be detected by people even in the field [35,36]. Once ignited, they are particularly difficult to extinguish and can persist for long periods of time (months, years), spreading deep into the ground and over extensive areas. Recent studies have quantified the maximum soil moisture content that allows smouldering spread for different types of [22, 23, 24, 25], the conditions for water suppression [37,38] and the creation of firebreaks [23]. For most peatland megafire, firefighting with water is very challenging because of the large amounts of water needed. Despite these laboratory studies, there is still a lack of complete understanding of the required water quantity to extinguish a peat fire. Building the trench as a firebreak is an effective way to stop their spread (Figure 2b), especially where water resources are limited.

## Pollution and health

**Figure 3:**
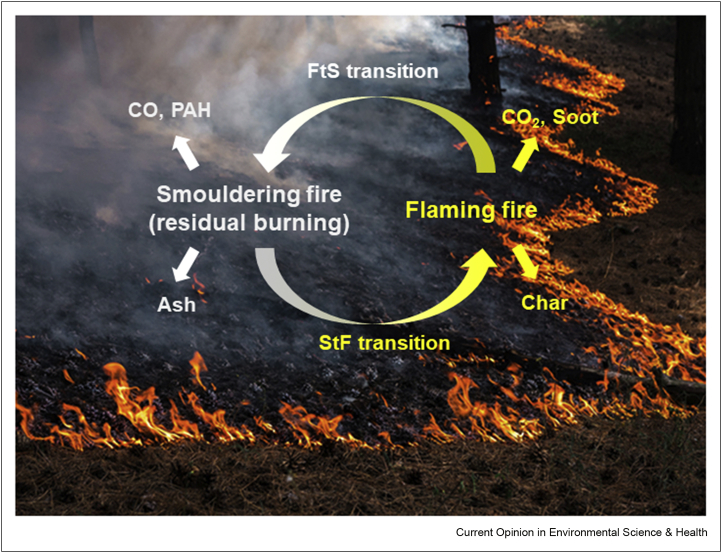
Visual and chemical characteristics of smouldering and flaming fires, and the transition processes from flaming to smouldering (FtS), and from smouldering to flaming (StF). After the flames die out, smouldering combustion of the char and the thicker fuels can continue burning biomass for long periods of time.

Smouldering emissions are a health concern for two reasons, generating large volumes of smoke and smoke toxicity. We have discussed the very large size of peatland fires that produce significant pollutants, whereas most other forest wildfires also result in pollution from smouldering. Smouldering of forest biomass can linger for days or weeks after flaming has ceased (Figure 1, Figure 3). Such a process is the transition from flaming to smouldering [2,35], and it is often referred to as residual burning. Smouldering residual burning results in large quantities of biomass consumed and a significant fraction of the total pollutants emitted into the atmosphere during a wildfire. In fact, many smoke management problems in the US associated with prescribed fires involved smouldering emission [39].

Biomass fuels that are prone to smouldering during wildfires include stumps, snags, downed logs, large branches, duff, roots, and organic soils. These fuels are characterised by having a significantly greater thermal time than fine fuels and favour the slow-burning of smouldering combustion. In forests, large quantities of fuel prone to smouldering are present on the surface and the ground, where most of the energy is released in smouldering wildfires. It has been reported that smouldering can consume 50% or more of the biomass in temperate and boreal wildfires [40], as well as in Amazonian tropical-woodland fires.

Because the combustion in the smouldering process is incomplete, the smoke contains higher levels of CO, CO_2_, NH_3_, and particle matter (PMs) [39,41], posing a significant health risk to population and emergency personnel [42,43]. Smouldering produced 130% more CO and 670% more hydrocarbons but 15% less CO_2_ and no NO_x_ [40]. Smouldering peat releases a large quantity of volatile organic compounds (VOCs) that are responsible for the large-scale and long-term haze events. Among volatile organic compounds, polycyclic aromatic hydrocarbons (PAHs) are well-known carcinogens, mutagens and teratogens. The exposure to pollutants during haze events (particularly particle matter and polycyclic aromatic hydrocarbons) results in various deleterious physiological responses, predominantly to the respiratory and cardiovascular systems.

Moreover, peat fires release Mercury (Hg) into the atmosphere at a rate 15 times greater than the flaming fires, and Hg is known for its nervous system toxicity on humans and downstream impacts on food chains [39,44]. During the 1997 peat fire and haze event, 16,400 Indonesian infant and foetal deaths were likely attributable to this haze pollution [45]. Thousands of excess deaths have been reported regularly after each haze event in Southeast Asia [46]. In Europe, Moscow recently suffered haze events in the summers of 2010 and 2012 from peat-megafire burning over several months, which increased the mortality rate by 1.6 times [16].

## Carbon emissions and climate change

**Figure 4:**
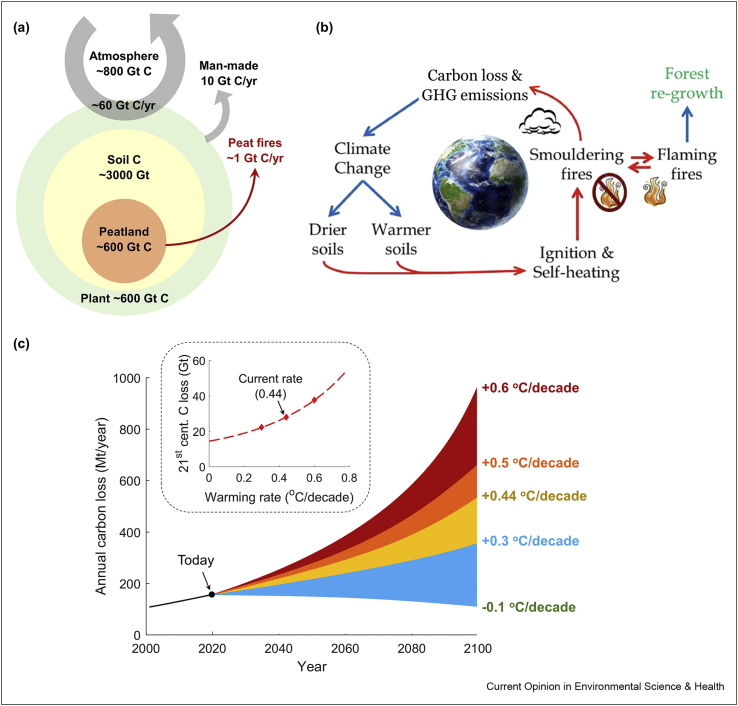
Diagrams of (**a**) the organic carbon distribution and flows on Earth surface, including the contribution of peat fires (modified from the study by Lehmann and Joseph [47]), (**b**) the positive feedback of smouldering wildfires on climate change [2], and (**c**) predicted effect of global warming on the carbon emission from the artic-boreal peat fires [11].

The 1997 and 2015 extreme haze events in Southeast Asia were caused by the spread of smouldering megafire in Indonesia peatlands during the El Niño climate event. Southeast Asia continues to be hit by haze on average once every three years. Carbon emissions from peat fires are important because peatlands, the peatland made by the natural accumulation of partially decayed biomass, is one of the largest reserves of terrestrial organic carbon (around 600 Gt). It represents 1/5-1/3 of the world's soil carbon and is comparable to the carbon stored in the atmosphere or living plants (Figure 4a) [44,47].

The soil carbon loss from peat fires can be estimated based on the depth of burn [48] and the burned area [49]. The depth of burn can be measured in the field, such as by drilling soil profile [8,50] and LIDAR remote sensing [51]. Satellite images (GIS data) are the most commonly used approach to assess the entire burned area [8,51,52]. However, the optical satellite systems may be severely hampered owing to residual haze after the fires, so there is considerable uncertainty in estimating the carbon emission from large-scale peat fires [8,53,54].

It is estimated that during the 1997-98 El Niño event, the carbon emission (0.81–2.57 Gt) from Indonesian peat fires is equivalent to 13–40% of that from the annual burning of fossil fuels [8,55]. The 2015 El Niño event also caused a massive burning of around 8000 km^2^ peatlands in Indonesia, which released 0.35–0.60 Gt carbon based on satellite data [54]. The global estimate for the greenhouse gases released by peat fires worldwide can be equivalent to >15% of man-made emissions [52]. This amount is comparable to the man-made emissions attributed to the entire European Union.

Their impact on climate is such that according to the Intergovernmental Panel on Climate Change (IPCC), fire emissions from organic soils such as peat and thawing permafrost are a key uncertainty in global carbon budgets. Moreover, the release of peat's ancient carbon creates a positive feedback mechanism in the climate system, a self-accelerating process (Figure 4b). Warmer climates at a global scale would result in more frequent and extensive smouldering fires worldwide in areas where warmer and drier histosols are found.

Warmer histosols and higher moisture deficiency create and accelerate smouldering hotspots. This is because smouldering ignition probability and self-heating are strong functions of soil moisture and soil temperature [2,11]. The latest estimation showed that under the warming rate of 0.44 °C/decade, carbon loss from the boreal peat fires on a warmer soil layer may increase from 143 Mt in 2015 to 544 Mt in 2100 and reach a total of 28 Gt for the 21st century (Figure 4c) [11]. In addition, more frequent flaming wildfires are predicted worldwide under warmer climates [56] and will lead to a more frequent transition to smouldering and residual burning. These lead to the burning of more ancient carbon, closing the loop when the climate warms up and dries more histosols [7].

## Arctic wildfires

**Figure 5:**
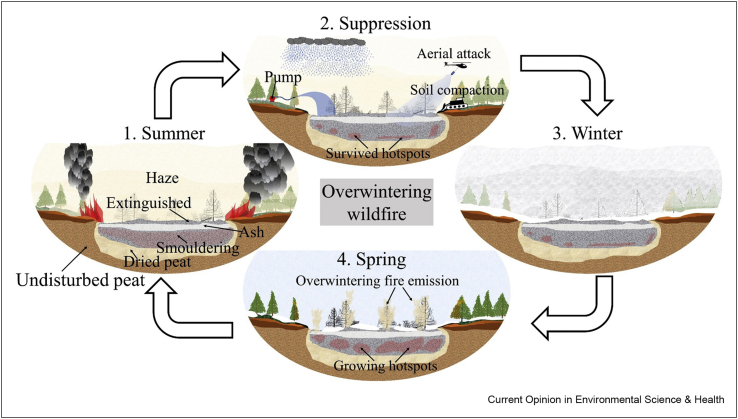
The four stage of an overwintering wildfire caused by smouldering combustion of histosols in the Arctic (by Santoso 2021, CC-BY).

Arctic wildfires increased in 2020 by 35% from the previous year and caused record-breaking carbon emissions from 66 Mt to 143 Mt carbon released to the atmosphere [9,11,57]. In the winter of cold regions like the Arctic and boreal, smouldering fires can still spread in soil layers and remain undetected as hotspots that are covered by snow, and laboratory experiments have demonstrated that smouldering peat fire can even survive below −35 °C [11]. This was recently confirmed by satellite observations [6]. These overwintering wildfires undergo four stages, as illustrated in Figure 5. In the warmer summer, both flaming and smouldering are part of the wildfire. The flames of the wildfires will be extinguished by rainfall, cold weather or firefighting. Nevertheless, smouldering hotspots can survive deep in histosols and not be quenched by water or winter because of the insulation effect of the topsoil and snow cover. In the Spring, the overwintering fires can grow in size and spread rate, helped by the dry conditions and warmer temperatures. They can even flare up on the surface again near the location where the last year's wildfires were extinguished.

The Arctic is already warming faster than the global average [58]. Average soil temperatures are increasing, and the thawing of permafrost is accelerating. This makes organic matter in the soil vulnerable to wildfires for the first time in a very long time, even millennia [18,59, 60, 61, 62]. The burning of this histosol in a smouldering wildfire would release ancient carbon, contributing to climate change (Figure 4b) and deteriorating the ecosystem [60,63,64]. It is predicted that no permafrost would remain if global warming reaches 6 °C [62,65], and the Arctic smouldering fires in the summer and overwinter would be accelerating this process [59].

## Future perspectives

Smouldering combustion is an important part of wildfires, especially in peatland fires, forest residual burning, and firebrand ignition. Smouldering wildfires increase mortality and economic losses, damage ecosystems and emit much carbon.

**Figure 6:**
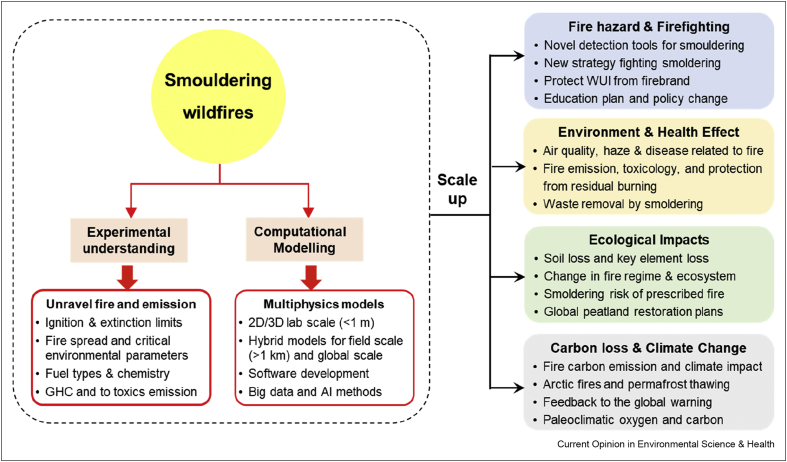
Diagram of the approaches and aim of future research to mitigate peat fires.

Of all the smouldering hazards, the millions of people who could be exposed to haze from peat fires, special in Southeast Asia and Siberia, need the most attention. Bad haze events have happened and will continue happening unless action is taken. The ambition should be to reduce the burden of smouldering fires and mitigate their hazards. We envision international and collaborative efforts that will enable new mitigation technologies for this overlooked problem. Some of these efforts are highlighted in Figure 6.

Once ignited, peat fires are particularly difficult to extinguish and could persist for long periods of time (weeks to months), spreading into the ground and over extensive areas. Water suppression could be effective to stop small fires but it does not work with large peat fires, which require very large amounts of water. Despite extensive fire-fighting attempts in hundreds of smouldering wildfires around the globe, very few fires have been successfully extinguished by human intervention; the most frequent outcome is burnout or natural flooding. However, partial flooding or removal of a massive portion of soil in a remote location is often not viable or not desirable. Smothering of deep-seated fire has been attempted, but it requires a very long holding time (months of continuous application) and hence is vulnerable to typical sealing failures.

However, the best way to mitigate fires is prevention, for smouldering as well. This is the active avoidance of fires from igniting. For example, keeping organic soils moist, avoiding drainage, and avoid ignition sources near dry land. When prevention fails, detection, monitoring and suppression become the priority (in this order). An important enabler for new technologies would be the up-scaling of laboratory findings to the field scale and target the real phenomenon.

Little is being performed on developing mitigation strategies. Many current monitoring and suppression technologies for smouldering fires are costly, rudimentary and inefficient. Often, know how comes from dealing with flaming wildfires, and unfortunately, this does not work for smouldering fires. We need a fundamental understanding of the phenomena to drive innovation and overcome this technological barrier.

Carbon emissions from peat wildfires have attracted scientific and policy attention over the last years. The IPCC included their contribution to global carbon emission and continues updating the calculation methods [68,69]. Nevertheless, current global estimations are extrapolated from limited field measurements, and therefore there is significant uncertainty in those numbers. Most probably, the IPCC is underestimating the contribution of peat fires because smouldering is much harder to detect than flaming, and many satellites sensing cannot distinguish one from the other. The most recent call for attention is coming from the Arctic and boreal regions, in which growing wildfires could be part of the positive feedback mechanism between smouldering and climate change, accelerating permafrost thaw. More studies and more clarifications are needed in all areas of smouldering wildfire.

Moreover, we claim that fighting the largest fires on Earth is an engineering task at the planet scale, and hence it is geoengineering (engineering on the Earth system). The Oxford Dictionary defines geoengineering as ‘the deliberate large-scale manipulation of an environmental process that affects the earth's climate, in an attempt to counteract the effects of global warming’. We propose to do geoengineering at a low level of geo-intervention aiming at providing technology and engineering solutions to the problem.

## Declaration of competing interest

The authors declare the following financial interests/personal relationships which may be considered as potential competing interests: Guillermo Rein reports financial support was provided by European Research Council. Xinyan Huang reports financial support was provided by National Natural Science Foundation of China.
